# Oxidative Stress in Pancreatic Beta Cell Regeneration

**DOI:** 10.1155/2017/1930261

**Published:** 2017-08-03

**Authors:** Jingjing Wang, Hongjun Wang

**Affiliations:** Department of Surgery, Medical University of South Carolina, Charleston, SC 29425, USA

## Abstract

Pancreatic *β* cell neogenesis and proliferation during the neonatal period are critical for the generation of sufficient pancreatic *β* cell mass/reserve and have a profound impact on long-term protection against type 2 diabetes (T2D). Oxidative stress plays an important role in *β* cell neogenesis, proliferation, and survival under both physiological and pathophysiological conditions. Pancreatic *β* cells are extremely susceptible to oxidative stress due to a high endogenous production of reactive oxygen species (ROS) and a low expression of antioxidative enzymes. In this review, we summarize studies describing the critical roles and the mechanisms of how oxidative stress impacts *β* cell neogenesis and proliferation. In addition, the effects of antioxidant supplements on reduction of oxidative stress and increase of *β* cell proliferation are discussed. Exploring the roles and the potential therapeutic effects of antioxidants in the process of *β* cell regeneration would provide novel perspectives to preserve and/or expand pancreatic *β* cell mass for the treatment of T2D.

## 1. Introduction

The prevalence of diabetes mellitus is increasing at an astounding rate worldwide. According to the WHO, the global prevalence of diabetes in 2014 was estimated to be 9% among adults aged 18 years and older. In 2012, an estimated 1.5 million deaths were directly caused by diabetes, and it was projected that diabetes will be the 7th leading cause of death in 2030. Although the etiology differs in the three major types of the disease—type 1 diabetes, type 2 diabetes, and gestational diabetes, all feature a crucial pathological change in the progression of diabetes, which is insufficient numbers of *β* cells to meet metabolic demand to control blood glucose levels. Pancreatic *β* cells, located in the islet of Langerhans, are essential for the maintenance of glucose homeostasis via the sensing of elevated blood glucose level and the subsequent production of glucose-lowering hormone insulin. Beta cell regeneration (neogenesis and proliferation) during the neonatal period is critical for the generation of sufficient pancreatic *β* cell mass/reserve and has a profound impact on long-term protection against T2D [1]. Moreover, under circumstances such as pregnancy or insulin resistance in T2D, enhanced *β* cell proliferation is present in response to the increased demand of insulin [2]. It is well-established that in response to hyperglycemia in diabetogenic states, *β* cell proliferation is substantially upregulated to various extents as a compensatory approach before the eventual loss of *β* cells' mass in later stage of diabetes [2, 3]. Thus, the need for *β* cell mass to be closely regulated under physiological and pathophysiological conditions on cell replication, size, apoptotic elimination, and, sometimes, neogenesis from progenitor cells is very important.

**Figure 1:**
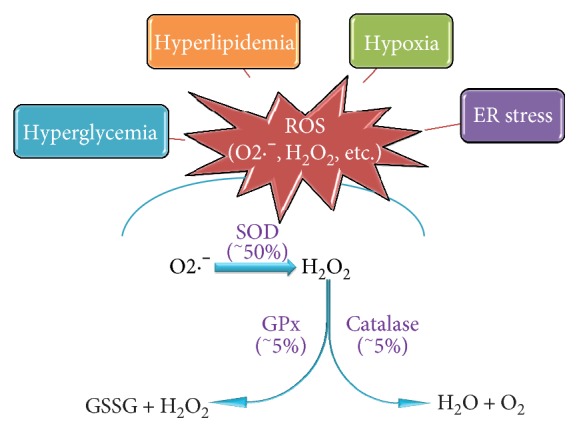
*β* cells are extremely susceptible to oxidative stress. Two major factors render *β* cells prone to the risk of oxidative stress: a high endogenous generation of ROS induced by stimuli including hyperglycemia, hyperlipidemia, hypoxia, ER stress, and low expressions of essential antioxidant enzymes such as SOD, catalase, and GPx. Percentages refer to the amount of mRNA expression in pancreatic islets versus liver tissue in rats.

In T2D, the pathogenic effect of high glucose, possibly accompanied with excessive amount of fatty acids in the case of obesity, is exhibited to a significant extent via imbalanced redox status, through the increased production of reactive oxygen species (ROS) and reactive nitrogen species which results in oxidative stress. Numerous studies observed elevated levels of oxidative stress markers in patients with T2D [4, 5]. Indeed, because of the high demand of insulin, *β* cells are among the most metabolically active tissues and highly rely on oxidative phosphorylation for the generation of adenosine triphosphate (ATP). Moreover, high oxygen consumption is a key factor for insulin secretion, especially in response to elevated blood glucose levels [6], which renders *β* cells to higher risk of ROS production and oxidative stress. On the other hand, *β* cells are particularly vulnerable to oxidative stress majorly due to the lack of antioxidant enzymes (Figure 1), which further weakened the ability of *β* cells in defense against oxidative stress.

A number of outstanding review articles have discussed the deleterious effects of oxidative stress on *β* cell death and dysfunction. During the past two decades, a plethora of evidence showed that oxidative stress is present in *β* cells while cell growth is most active and tightly controlled, such as during embryogenesis and pathological progressions of obesity and diabetes. These data indicate an important role of oxidative stress in *β* cell regeneration. Therefore, in this review, we focus on summarizing recent studies reporting the impacts of oxidative stress on *β* cell regeneration. As such, we do not discuss the impacts of oxidative stress in *β* cell apoptosis and function. We first overview the susceptibility of *β* cells to oxidative stress, as well as the molecular mechanisms of *β* cell regeneration. We then focus on describing recent studies reporting various effects of oxidative stress on *β* cell regeneration, to deepen our understanding on the broad impacts of oxidative stress on *β* cells.

## 2. Pancreatic *β* Cells Are Extremely Sensitive to Oxidative Stress

Aerobic cells produce ROS such as superoxide anion (O2·^−^) and H_2_O_2_ during oxidative phosphorylation in the mitochondria as by-products [7, 8]. Like in other aerobic cell types, mitochondrial electron transport is the main source of superoxide anions of pancreatic *β* cells. Superoxide anion is a reactive molecule, but it can be converted to H_2_O_2_ by superoxide dismutase (SOD) isoenzymes and then to oxygen and water by enzymes including catalase (CAT), glutathione peroxidase (GPx), and peroxiredoxin (Prx). Beta cells have lower antioxidative enzymes to combat the continuously generated superoxide anions. They are only equipped with about 50% of the SOD and 5% of H_2_O_2_-scavenging enzymes GPx and CAT compared to those enzymes found in the liver (Figure 1) [9]. This makes *β* cells highly sensitive to ROS-related signaling and distinctively susceptible to oxidative and cytotoxicity stress. Indeed, a substantial amount of evidence supports the notion that intrinsically low levels of antioxidant activity of islets render them particularly at risk for ROS-induced damage [10–13].

Several conditions leading to ROS generation in *β* cells have been proposed, among which are hyperglycemia, hyperlipidemia, hypoxia, and endoplasmic reticulum (ER) stress (Figure 1). Hyperglycemia, defining diabetes, can be directly associated with increased ROS generation through a variety of mechanisms [13]. During chronic hyperglycemia, *β* cells are exposed to high glucose concentrations for an extended period of time. In this context, the normal route of glycolysis gets saturated and excess glucose is shunted towards alternative ROS-forming pathways including glycosylation (Schiff reaction) [14], glucose autoxidation [15, 16], and glucosamine pathway [17], all of which lead to the accumulation of ROS and induction of oxidative stress.

In addition to hyperglycemia, exposure to excessive lipid (hyperlipidemia) has also been shown to activate cell stress responses including oxidative stress, which contributes to lipotoxicity in *β* cells in T2D [18]. An *in vitro* study using prolonged exposure to free fatty acid (FFA) exhibited increased islet ROS production in mitochondria, which was prevented by overexpression of the enzyme, GPx4 [19]. The mechanism by which FFA promotes ROS generation in mitochondria remains unclear. One possible explanation is the activation of nicotinamide adenine dinucleotide phosphate (NADPH) oxidase as evidenced by Morgan et al., who reported an increase of the p47 (phox) component of the NADPH oxidase and subsequent production of ROS in *β* cells after 24 hours incubation with palmitic acid [20]. Koulajian et al. further reported that p47 (phox)-null mice or the treatment of an NADPH oxidase inhibitor protected islets from oleate-induced increased ROS production [21]. Another mechanism that contributes to lipid-induced oxidative stress in *β* cells is the modulation of respiratory chain by FFA. *β* cells exposed to FFA exhibited increased ROS production, and respiratory complex I in mitochondria seemed to be the major radical source [22].

In *β* cells, hypoxia is induced when the cells are exposed to high glucose, with significant upregulations of several hypoxia-related genes including adrenomedullin (*Adm*) and pyruvate dehydrogenase kinase 1 (*Pdk1*) [23]. In addition, prediabetic Zucker rat islets showed increased expression of hypoxia-related genes along with a severely disturbed vascular integrity, strongly suggesting the presence of hypoxia in islets in the development of T2D [24]. It is well-established that hypoxia or low oxygen tension could paradoxically lead to increased ROS generation, majorly within complexes I and III of the mitochondrial electron transport chain [6, 25], making hypoxia a resource for ROS presented in pancreatic *β* cells.

ER stress is also closely entwined with oxidative stress, especially in insulin-producing *β* cells. ER stress occurs when the level of misfolded protein, mostly proinsulin in the case of *β* cells, exceeds ER adaptive capabilities which leads to a panel of signaling events leading to reduced insulin transcription and translation [26]. Hydrogen peroxide is generated as a by-product during the formation of oxidized protein in the ER, which is an important source of cellular ROS. Furthermore, under the condition of ER stress, the accumulation of dysregulated disulfide bond formation and breakage results in an excessive amount of ROS which also causes oxidative stress [27]. Additionally, ER stress activates C/EBP homologous protein (CHOP) that is demonstrated to contribute to the induction of oxidative stress during ER stress [28]. In summary, these data suggest that ROS is continuously generated in *β* cells at a considerable amount, addressing the significance of understanding the impacts of ROS and oxidative stress on *β* cells.

## 3. *β* Cell Neogenesis and Replication

**Figure 2:**
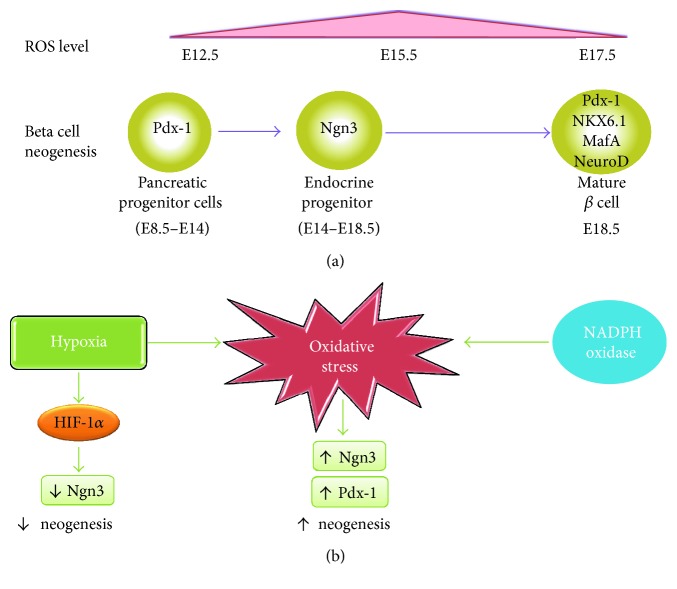
Effects of oxidative stress in *β* cell neogenesis. (a) An overview of the ROS expression levels at distinct stages of *β* cell development in mouse embryo. The presence of ROS (E12.5–E17.5) largely overlaps with the Ngn3 expression period (E14–E18.5). (b) Schematic diagram of effects of ROS on *β* cell neogenesis. On the one hand, hypoxia as an important inducer of ROS activates HIF-1*α* which suppresses Ngn3 expression, which results in impaired *β* cell neogenesis. On the other hand, ROS directly upregulates Ngn3 and Pdx-1 in *β* cell development, and NADPH oxidase seems to be a crucial source of ROS in this process, leading to increased β cell neogenesis.

Pancreas provides new *β* cells through neogenesis (development of *β* cells from non-*β* cell precursors) and through replication (mitotic division) of differentiated *β* cells. Beta cell neogenesis occurs mostly before birth. The neogenesis begins from a pool of pancreatic progenitor cells that express transcription factor pancreatic duodenal homeobox-1 (Pdx-1) starting at embryonic day 8.5 (E8.5) in mice. Later on, a subset of cells that transiently express the transcription factor neurogenin-3 (Ngn-3) between E14 and E18.5 (endocrine progenitor cells) marks the onset of endocrine cell differentiation [29]. Mature *β* cells eventually express high levels of transcriptional factors including Pdx-1, Nkx6.1, MafA, and NeuroD (Figure 2(a) [30, 31]. The fastest expansion of the *β* cell mass occurs in late fetal gestation. In rat fetus, *β* cell population was estimated to be doubled every day starting from 16 days postconception [32]. Similarly, in human fetal pancreas, rapid expansion of the *β* cell mass has been noted from 20-week fetuses [33]. Although *β* cell number is usually considered to be stable after birth, accumulating evidence indicates the presence of postnatal *β* cell replication. Meier et al. observed a 7-fold expansion of *β* cell mass in human from birth to adulthood [34], and the major mechanism for this is attributable to *β* cell replication, rather than neogenesis, which is consistent with studies in mice [35]. Beta cell replication rates in adult islets decline substantially (from ~20% per day in pups to ~2% in early adulthood [36] and then approaches 0 in aged animal [37]). Despite the very slow turnover rate of *β* cells, plenty of evidence indicates that *β* cell is capable of regeneration under certain physiological or pathological conditions in adults, such as pregnancy, obesity, or diabetes, to meet increased metabolic demands. Significantly, increased number of *β* cells is observed in response to elevated demand of insulin in several rodent models of obesity and diabetes, and the main mechanism underlying this is the proliferation of fully differentiated *β* cells [2, 38, 39].

## 4. The Role of Oxidative Stress in *β* Cell Neogenesis

Growing evidence indicates that low oxygen tension or hypoxia controls the stemness and lineage commitment of several precursor cell types during development, including pancreatic *β* cells. Recently, Liang et al. observed a dynamic change of ROS levels in pancreas development, where ROS was detected in mouse embryonic pancreas as early as E12.5 and peaked at E15.5 (Figure 2(b)) [40]. By culturing embryonic pancreatic cells under controlled O_2_ concentrations, researchers reported that *β* cell neogenesis is positively controlled by oxygen tension in a dose-dependent manner through hypoxia-inducible factor- (HIF-) *α*. They found that repressed HIF-1*α* expression led to elevated development of Ngn3-positive endocrine progenitors, which resulted in increased *β* cell neogenesis and development [41]. The involvement of hypoxia-induced HIF-1*α* in Ngn3 expression and *β* cell neogenesis was also confirmed in both human and mice embryonic pancreas [42]. The deletion of von Hippel-Lindau (VHL), a gene encoding a protein necessary for the proteasomal degradation of HIF-1*α*, led to impaired *β* cell development, which further confirmed the negative impact of HIF-1*α* on *β* cell neogenesis [42]. Interestingly, although oxidative stress was observed in embryonic pancreas under hypoxia condition (3% pO_2_), it seemed to have little effect on hypoxia-induced Ngn3 suppression (Figure 2(b)) [42].

Despite the suppressive effect of hypoxia on *β* cell neogenesis, emerging evidence indicates that ROS by themselves could stimulate *β* cell regeneration. The inhibition of NADPH oxidase, which is the major source of endogenous ROS, reduced the expression of markers for endocrine cell differentiation including Ngn3 and impaired the differentiation of endocrine progenitors in cultured pancreatic rudiments. Addition of exogenous ROS reversed this effect, which further confirmed that Ngn3 expression is ROS-dependent. Newborn rats exhibit spontaneous remission of *β* cell shortly after streptozotocin- (STZ-) induced damage. Interestingly, the administration of NADPH oxidase inhibitor attenuated *β* cell regeneration after STZ injection in rat pups, supporting a positive effect of NADPH oxidase-derived ROS on progenitor cell differentiation [40]. In accordance with this, mouse pancreatic explant treated with angiotensin (1–7) resulted in an increased level of ROS, which is accompanied with elevated expression Ngn3. This was also blocked by the presence of an NADPH oxidase inhibitor [43]. Moreover, Hoarau et al. reported that H_2_O_2_ enhances the *β* cell neogenesis in rat embryonic pancreas, and the treatment of pregnant rats with an antioxidant agent, N-acetyl-cysteine (NAC), decreases *β* cell differentiation in their progeny. They proposed that extracellular signal-regulated kinases 1/2 (ERK1/2) pathway was activated by H_2_O_2_ and plays a critical role in ROS-induced *β* cell neogenesis [44]. Recently, Sun et al. discovered that hypoxia condition (2% pO_2_) effectively directs mesenchymal stem cell (MSC) differentiation into early *β* cell progenitors that were further induced into insulin-producing *β* cells. They detected significantly higher levels of *β* cell differentiation markers Pdx-1 and HNF6 in MSCs treated with hypoxia compared with the normoxia group; however, Ngn3 expression seemed to be impaired under this condition (Figure 2(b)) [45].

## 5. Oxidative Stress Influences Cell-Cycle Regulators in *β* Cell Proliferation

**Figure 3:**
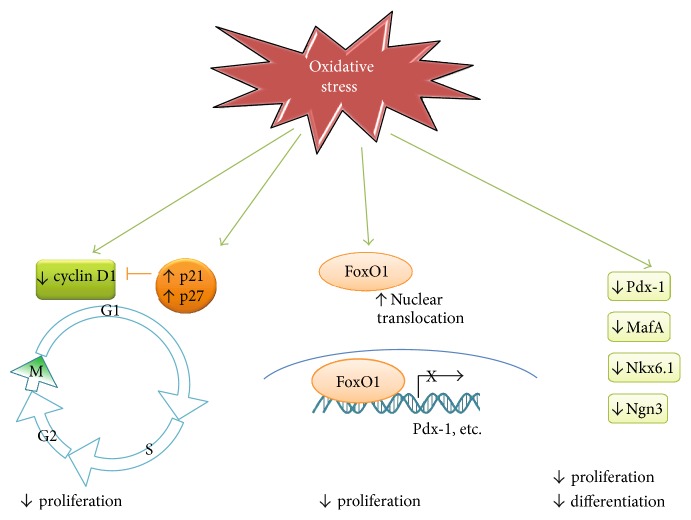
Oxidative stress plays critical roles in *β* cell cycle. On cell-cycle regulators, accumulated ROS in beta cells suppressed the expression of cyclins D1 and D2, as well as increased cell-cycle inhibitors such as p21 and p27, leading to decreased *β* cell proliferation rate. Meanwhile, ROS promoted nuclear the translocation/activation of FoxO1, which in turn prevents *β* cell replication through the inhibition of Pdx-1 and possibly other *β* cell-related gene transcriptions. Furthermore, ROS also directly downregulates transcription factors such as Pdx-1, MafA, Nkx6.1, and Ngn3 that are crucial in beta cell proliferation and differentiation.

Like in other cells in mammals, cell cycle in *β* cells is governed intracellularly by various complexes of cyclins and cyclin-dependent kinases (CDKs) and by their inhibitors. Cyclins D1 and D2 are proposed to be essential for postnatal *β* cell growth in rodents and regulate compensatory *β* cell replication in response to excessive nutrient or insulin resistance [35, 46, 47]. CDK inhibitors, such as the Cdk-interacting protein (CIP)/the kinase inhibitor protein (KIP) families including p21 and p27, are involved in cyclin binding and kinase inhibitory function. A high-throughput RNAi screening strategy demonstrated that silencing p21 facilitated the cell-cycle entry of quiescent adult human pancreatic *β* cells. Overexpression of p27 in *β* cells causes hyperglycemia by four weeks of age and markedly reduced islet mass by 8 weeks of age [48]. These studies demonstrated critical roles of p21 and p27 in *β* cell proliferation. However, how these cell-cycle regulators are modulated in *β* cells remains unclear. Emerging evidence suggests that oxidative stress may play a role in regulating cell cycle (Figure 3). Kaneto et al. reported that H_2_O_2_-induced oxidative stress significantly elevated p21 mRNA in rat islets. Notably, they also found that p21 mRNA increased as hyperglycemia becomes evident in Zucker diabetic fatty (ZDF) rats *in vivo* [49]. In line with this, Zhang et al. observed that intermittent high glucose induced an elevation of intracellular ROS production, leading to decreased cyclin D1 expression as well as increased p21 and p27 expression, and resulted in significantly reduced proliferation rate in the INS-1 *β* cell line [50]. Nuclear receptor subfamily 2 group E member 1 (NE2E1), an essential regulator of the growth of neural stem cells, has been reported to regulate *β* cell proliferation. Knockdown of NE2E1 in another *β* cell line, the MIN6 cells, resulted in decreased proliferation with a partial G0/G1 cell-cycle arrest. Interestingly, NE2E1 deficiency also led to decreased antioxidant enzymes and an augmentation of palmitate-induced oxidative stress in *β* cells, suggesting a potential role of imbalanced oxidant/antioxidant system in the regulation of *β* cell proliferation [51].

On the contrary, intermittent hypoxia, which mimics the hypoxic stress present in obstructive sleep apnoea, seemed to trigger *β* cell proliferation. Yokoe et al. found that mice exposed to intermittent hypoxia exhibited substantially higher proliferation rate in their pancreatic *β* cells compared with those in the normoxia group [52]. Similarly, Xu et al. applied intermittent hypoxia and observed a significant elevation of *β* cell proliferation both *in vitro* and *in vivo*, which coincided with increased cyclin D2 translocation to the nucleus. However, overexpression of MnSOD did not affect the mitogenic effect of intermittent hypoxia [53]. Nonetheless, the molecular mechanism underlying intermittent hypoxia-induced *β* cell proliferation is still indefinite. The experimental differences between intermittent hypoxia and other induction methods of oxidative stress (H_2_O_2_ treatment or high glucose as mentioned above) may help explain these seemingly contradictory findings.

## 6. Effects of Oxidative Stress on Transcription Factors for *β* Cell Regeneration

Forkhead box class O family member proteins (FOXOs) are transcription factors that play important roles in *β* cell differentiation, proliferation, and survival [54]. Among them, FoxO1 is the most predominantly expressed FoxO factor in isolated mouse islets, the insulinoma cell line (*β*TC3 cells) and human islets [54]. FoxO1 negatively regulates *β* cell proliferation and differentiation through two mechanisms by the modulation of Pdx-1 gene transcription and nuclear translocation. Firstly, FoxO1 functions as a repressor to compete with the transcription factor FoxA2 for binding to Pdx-1 promoter, leading to the reduction of Pdx-1 transcription and impaired *β* cell growth [55]. Secondly, the nuclear translocation of FoxO1 accompanies with the nuclear exclusion of Pdx-1, which further suppresses *β* cell proliferation [55]. In contrast to its inhibitory effects on *β* cell proliferation, FoxO1 seems to exert a protective effect against oxidative stress-induced damage. It was proposed that FoxO1 facilitated cells that undergo a state of “premature senescence” to protect *β* cells against hyperglycemia-induced oxidative stress [56, 57]. In cultured *β* cells, FoxO1 is constitutively phosphorylated and remained inactivated in cytoplasm. Upon exposure to hydrogen peroxide, around 30% FoxO1 translocate into nuclei in *β*TC3 cells, which resulted in growth arrest [58]. Further studies showed that upon oxidative stress, FoxO1 formed a complex with the promyelocytic leukemia protein (Pml) and the NAD-dependent deacetylase sirtuin-1 (Sirt1), leading to upregulated NeuroD and MafA expression, both of which are important transcription factors for *β* cell development and Ins2 gene transcription. However, the tradeoff of Pml-mediated transcription activity of FoxO1 accelerated the degradation of this protein [58]. Therefore, it is obvious that the transient effects of FoxO1-induced upregulation of MafA and NeuroD are not sufficient to counteract the replication inhibition of FoxO1 nuclear translocation, resulting in the net effect of growth arrest under oxidative stress in *β* cells (Figure 2()).

In fact, MafA plays a pivotal role in regulating the replication and development of *β* cells [59] and is exceptionally sensitive to oxidative stress. Under H_2_O_2_-induced oxidative stress, MafA is inactivated and translocated to cytoplasm within 30 min. The inactivation of Pdx-1 and Nkx6.1, two transcription factors required for *β* cell differentiation, was observed upon H_2_O_2_ treatment. Importantly, levels of MafA and Nkx6.1 were also severely compromised in human T2D islets as well as hyperglycemic leptin-receptor-deficient (db/db) mouse islets. Further studies on transgenic db/db mice overexpressing the antioxidant enzyme Gpx1 showed that the nuclear MafA and Nkx6.1 levels were restored, confirming that loss of these important *β* cell transcription factors in T2D is oxidative stress-dependent [60]. Chiou et al. showed that overexpression of MafA facilitated placenta-derived mesenchymal stem cells to differentiate into mature insulin-producing cells and rendered those insulin positive cells more resistant to H_2_O_2_-induced oxidative stress [61].

As discussed previously, ER stress contributes to ROS generation and induction of oxidative stress in *β* cells. Studies have shown that ER stress also significantly impacts *β* cells' regeneration. Accumulating evidence suggests that protein kinase-R-like ER kinase (PERK) and its substrate eukaryotic translation initiation factor 2*α* (eIF2*α*), major components in ER stress, are critical in the control of *β* cell proliferation and differentiation [62–66]. Mutations in PERK (EIF2AK3) cause a complex genetic disorder of the Wolcott Rallison syndrome with permanent neonatal diabetes characterized by *β* cell depletion [67]. Mice lacking PERK or PERK-mediated eIF2*α* phosphorylation develop a similar phenotype with early onset diabetes and reduced *β* cell mass, accompanied with decreased expression of Pdx-1 and MafA [63–65]. Detailed studies suggest that in the absence of PERK-mediated eIF2*α* phosphorylation, islets/*β* cells exhibited to be under significant oxidative stress as indicated by peroxynitrite modification of tyrosine residues resulted from the reaction between superoxide and nitric oxide. It seemed that the defects of *β* cell proliferation and differentiation are partly attributable to uncontrolled oxidative stress in the absence of functional PERK/eIF2*α* pathway [63].

## 7. Effects of Antioxidants on *β* Cell Proliferation in T2D

Supplements with antioxidant properties have long been used to decrease oxidative stress and improved pancreatic *β* cell proliferation and function in T2D [68]. Selenium is a necessary trace element in the body that can act as an antioxidant nutrient in different cell types via incorporation of selenocysteine into selenoproteins through a UGA codon-encoded genetic process. Experimental data suggest that supplementation with selenium as an antioxidant could delay the development of T2D by decreasing oxidative stress [69]. Wang et al. found that long-term administration of dietary selenate supplementation to db/db mice suppressed the elevation of fasting glucose level. Further study showed that selenate supplementation significantly increased islet size, along with the upregulation of genes that encode proteins involved in *β* cell proliferation and differentiation, suggesting a positive role of antioxidant in the proliferation of *β* cells in the progression of diabetes [70]. Consistent with this study, Chang et al. discovered that a *Stigmata maydis* (corn silk) fraction exhibited antioxidant effect in hydrogen peroxide- or methylglyoxal-induced oxidative stress in a rat *β* cell line. They demonstrated that this fraction of corn silk attenuated the suppression of *β* cell proliferation induced by acute H_2_O_2_ treatment, indicating a potential benefit of proproliferation in *β* cells under oxidative stress in T2D [71]. Bitter gourd (*Momordica charantia* Linn.) is a type of well-established remedy food for diabetes patients. The boiled water extraction of sun-dried bitter gourd possesses remarkable free radical scavenging activity, and studies showed that the higher antioxidant activity of the fraction, a corresponding higher proliferation activity was observed on the treated *β* cells [72]. In addition, a number of natural phenolic compounds, including quercetin, catechin, and ascorbic acid, exhibited great antioxidant properties in various cells [73]. Interestingly, although in normal growth media, these phenolic compounds even amplified H_2_O_2_-induced proliferation inhibition in *β* cells at higher concentrations, it was demonstrated that this was caused by a quick generation of H_2_O_2_ from the incubation of these compounds with the media. Once this is eliminated, the growth inhibition of *β* cells induced by H_2_O_2_ treatment was efficiently suppressed by these compounds [74]. These observations strongly suggest that the enhancement of *β* cell proliferation contributes to the beneficial effects of antioxidants used in T2D.

## 8. Conclusion

Currently, there is considerable interest in targeting *β* cell regeneration as an effective approach to replenish insulin for patients with diabetes. Recent insights indicated that oxidative stress and *β* cell regeneration are highly interrelated biological process, not only demonstrated by the fact that they coexist under several physiological and pathological conditions but also reflected by the profound direct and indirect impacts of oxidative stress on *β* cell regeneration. The roles of oxidative stress in *β* cell neogenesis and proliferation are complicated by different stages of cellular growth and different stimuli of the stress. It seems that oxidative stress majorly exerts a negative effect on *β* cell development and proliferation. However, ROS seems to be temporarily required for *β* cell neogenesis in embryonic pancreas and during *β* cell replication shortly after birth. Importantly, several antioxidant supplements increased proliferation rates of pancreatic *β* cells, which not only confirmed the deleterious effects of oxidative stress on *β* cell regeneration but also suggested potential benefits of antioxidants as a therapeutic method for T2D patients, in respect of improving *β* cell regeneration. In summary, oxidative stress plays critical roles during pancreatic *β* cell neogenesis and proliferation, as well as during the development of T2D.

## Abbreviations

T2D: Type 2 diabetes
ROS: Reactive oxygen species
ATP: Adenosine triphosphate
SOD: Superoxide dismutase
CAT: Catalase
Gpx: Glutathione peroxidase
Prx: Peroxiredoxin
ER: Endoplasmic reticulum
FFA: Free fatty acid
CHOP: C/EBP homologous protein
Pdx-1: Pancreatic duodenal homeobox-1
HIF-1*α*: Hypoxia-inducible factor-1 *α*
STZ: Streptozotocin
CDK: Cyclin-dependent kinase
FOXO: Forkhead box class O family protein
PERK: Protein kinase-R-like ER kinase.
